# Impact of probiotic-supplemented water on the performance and physiological responses of broilers under normal and heat stress conditions

**DOI:** 10.14202/vetworld.2025.1059-1069

**Published:** 2025-04-30

**Authors:** Rahaf Istiteh, Mohannad Abuajamieh, Mohmmad Al-Qaisi, Mohamed A. Abedal-Majed, Anas Abdelqader

**Affiliations:** Department of Animal Production, School of Agriculture, The University of Jordan, 11942, Amman, Jordan

**Keywords:** blood metabolites, broiler chicken, heat stress, intestinal morphology, performance, probiotics, thermoregulation

## Abstract

**Background and Aim:**

Heat stress (HS) significantly compromises broiler performance, intestinal health, and immune responses, posing a growing threat under climate change. Probiotics (PROs) have been proposed as a nutritional intervention to mitigate HS effects, yet their efficacy through drinking water remains understudied. This study aimed to evaluate the effects of water-supplemented PROs on growth performance, physiological responses, intestinal morphology, and inflammatory biomarkers in broiler chickens under thermo-neutral (TN) and HS conditions.

**Materials and Methods:**

A total of 192 one-day-old Ross 308 broiler chicks were randomly allocated to four treatment groups (6 replicates/group): TN with control (CON) water, TN with PRO-supplemented water, HS with CON water, and HS with PRO-supplemented water. PROs (*Bacillus subtilis* and *Bacillus pumilus*) were administered in drinking water at 50 mg/L from day 1 to 35. Birds in the HS groups were subjected to 3 h daily heat exposure (33°C ± 2°C) from days 31 to 35. Growth performance, physiological indicators, intestinal histomorphology, and blood biomarkers were assessed.

**Results:**

HS significantly increased rectal temperature (Tr) (1.6°C; p < 0.01) and respiratory rate (57 breaths/min; p < 0.01). PRO supplementation reduced Tr by 0.17°C (p = 0.01) but did not affect performance metrics (feed intake, body weight gain, feed conversion ratio). HS reduced villus height (VH) and surface area in the jejunum and ileum (p < 0.05), while PROs partially ameliorated VH under HS. No significant effects of PROs were observed on serum amyloid A or tumor necrosis factor-alpha levels. However, PROs tended to reduce diamine oxidase levels (24%; p = 0.09). HS also decreased albumin and glucose levels (p ≤ 0.02).

**Conclusion:**

HS adversely affected intestinal integrity and selected blood metabolites. Although PRO supplementation had limited effects on performance and inflammatory biomarkers, it conferred modest thermoregulatory benefits and tended to improve intestinal permeability. Further research is warranted to optimize PRO formulation and assess synergistic strategies for HS mitigation in poultry.

## INTRODUCTION

The poultry sector is vital for providing high-quality animal-derived protein to the global food supply chain [1]. Within this sector, broiler chickens have undergone intensive genetic selection aimed at maximizing post-hatch growth rates and feed conversion efficiency. However, this has inadvertently heightened their vulnerability to environmental stressors, particularly extreme temperatures [2]. According to projections by the Organization for Economic Co-operation and Development/Food and Agriculture Organization, by 2030, meat will account for approximately 41% of the total protein derived from animal sources. Over the past 10 years, average global surface temperatures have risen by roughly 1.2°C, and a further increase of over 1.0°C is anticipated within the next three decades [3]. Such climatic trends pose considerable challenges to maintain optimal broiler productivity [4]. High ambient temperatures can detrimentally impact broiler performance by reducing feed intake (FI) and weight gain, disrupting physiological balance, and elevating mortality rates, thereby compromising both economic efficiency and animal welfare [5]. Heat stress (HS), a critical issue in poultry production, has been linked to increased mortality and substantial economic losses [6–9], with estimates indicating financial damages of approximately USD 2.36 billion in the U.S. livestock sector and more than USD 120 million in the poultry industry alone [10]. The intestinal mucosal barrier, a selectively permeable membrane, constitutes the first line of defense against luminal pathogens, antigens, and other noxious agents [11]. HS compromises gut health by impairing nutrient absorption, weakening immune defenses, and disrupting intestinal function [12, 13]. Increased intestinal permeability during HS facilitates the translocation of pathogenic microorganisms, which can trigger heightened systemic inflammatory responses [14].

Various nutritional strategies have been investigated to counteract the detrimental effects of elevated ambient temperatures in poultry production systems [14]. These interventions include midnight feeding, supplementation with vitamins, minerals, phytochemicals, probiotics (PROs), and prebiotics [15]. Among these, PROs have received substantial attention due to their beneficial impact on gastrointestinal health [14]. When administered at appropriate dosages, PROs can exert positive effects on host physiology by suppressing pathogenic bacteria and enhancing digestive efficiency and nutrient uptake [16, 17]. In addition, PROs have shown promise in mitigating the adverse effects of HS by improving gut architecture, maintaining microbial homeostasis, supporting physiological resilience, and modulating immune responses [18]. The application of PROs through drinking water is particularly favored by both producers and researchers due to its superior absorption efficiency compared to feed-based delivery. Furthermore, water-based administration simplifies handling and may reduce labor inputs [19]. PROs have also been shown to counteract HS-induced alterations in intestinal morphology in laying hens [5] and contribute positively to egg quality parameters such as egg weight and Haugh units [6]. In ruminants, such as goats, PRO supplementation has been demonstrated to alleviate HS effects by enhancing ruminal fermentation, improving nutrient digestibility, increasing volatile fatty acid output, and ultimately boosting performance [20].

Despite the well-documented adverse effects of HS on broiler performance, intestinal morphology, and immune function, effective and practical mitigation strategies remain limited. PRO supplementation has been explored as a potential intervention due to its capacity to enhance gut health, modulate immune responses, and improve nutrient utilization under thermal stress conditions. However, most studies have focused on in-feed delivery of PROs, while investigations into the efficacy of water-based administration – particularly under HS conditions – are comparatively scarce. Moreover, there is limited evidence concerning the use of specific strains such as *Bacillus subtilis* and *Bacillus pumilus* delivered through drinking water to modulate physiological and metabolic responses in heat-stressed broilers. The extent to which water-supplemented PROs can alleviate HS-induced physiological disturbances, intestinal damage, and inflammatory responses remain unclear, necessitating further investigation.

This study aimed to evaluate the effects of administering a PRO formulation (*B. subtilis* and *B. pumilus*) through drinking water on the performance, physiological responses, intestinal morphology, and inflammatory biomarkers of broiler chickens reared under thermo-neutral (TN) and HS conditions. Specifically, the research sought to determine whether water-based PRO supplementation could attenuate the negative impacts of HS and enhance broiler resilience, intestinal integrity, and productivity.

## MATERIALS AND METHODS

### Ethical approval

All experimental procedures were approved by the Animal Ethics Committee of the Deanship of Scientific Research at the University of Jordan (Approval no. 286/2023-2024).

### Study period and location

The study was conducted from October to December 2023 at the Animal Environmental Physiology Laboratory, School of Agriculture.

### Experimental design

**Figure 1:**
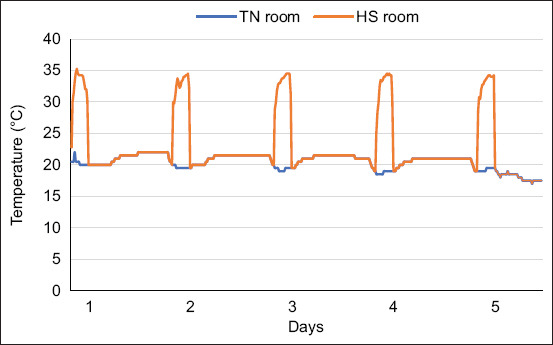
Ambient temperature (°C) in the thermo-neutral (TN) (20°C ± 2°C) and the heat stress (HS) (33°C ± 2°C) rooms during (d31–35).

A total of 192 one-day-old Ross 308 broiler chicks were procured from a commercial hatchery and randomly assigned to four treatment groups in a 2 × 2 factorial design (six replicates per treatment and eight chicks per replicate). The treatment groups were (1) TN control with tap water (TNCON), (2) TN with PRO-supplemented water (TNPRO), (3) HS control with tap water (HSCON), and (4) HS with PRO-supplemented water (HSPRO). Birds in the HS groups (HSCON and HSPRO) were exposed to cyclic HS (33 ± 2°C) for 3 h daily from days 31 to 35, a previously validated model simulating natural summer heat waves (Figure 1) [12].

### Animal management

Chicks were housed under two environmental regimens: TN (21 ± 2°C) and HS (33 ± 2°C). Broilers under TN conditions were maintained in three electrically heated brooders, each equipped with eight individual cages (0.75 × 0.75 × 0.35 m) providing optimal growth conditions. HS was induced in climate-controlled chambers (2.70 × 2.10 × 2.34 m) by gradually increasing the ambient temperature. Environmental variables, including temperature and ventilation, were monitored regularly to ensure accuracy. A continuous 24-h lighting schedule was implemented to mimic commercial poultry management practices.

### Dietary program

Chicks were fed *ad libitum* with standard starter, grower, and finisher diets throughout the experiment (Table 1). During the heat challenge period, birds in HS chambers experienced temporary restrictions in feed and water access.

**Table 1:** Feed ingredients and nutrient compositions used in the experiment.

Ingredients	Unit	Starter (1–11 days old)	Grower (12–22 days old)	Finisher (23–36 days old)
Corn	kg	581	626	662.00
Soybean meal	kg	375	325	280
Soy oil	kg	5	12	22
Calcium carbonate	kg	14	12	11
Vitamin and minerals premix 2.5%^1^	kg	25	25	25
Total		1000	1000	1000
Calculated analysis				
Dry matter	%	88	88	88
Metabolizable energy	Kcal	3000	3100	3200
Crude protein	%	22.40	20.50	18.70
Calcium	%	0.96	0.87	0.79
Available phosphorus	%	0.48	0.44	0.40
Digestible lysine	%	1.32	1.18	1.08
Digestible methionine + cystine	%	1.00	0.92	0.86
Digestible threonine	%	0.88	0.79	0.72
Digestible valine	%	1.00	0.91	0.84
Sodium	%	0.16	0.16	0.16
Chloride	%	0.20	0.20	0.20

1 Premix provided the following (per kg diet): 120,000 IU of Vitamin A, 3,500 IU of Vitamin D3, 40 IU of Vitamin E, 2.5 mg of Vitamin B1, 8 mg of Vitamin B2, 5 mg of Vitamin B6, 150 µg of riboflavin, 30 µg of B12, 1.5 mg of folic acid, 45 mg of niacin, and 13 mg of pantothenic acid, 60 mg of Fe, 10 mg of Cu, 80 mg of Mn, 80 mg of Zn, 1 mg of I, and 0.2 mg of Se

### PRO supplementation

PROs (NOVELA ECL+^®^, United Animal Health Inc., USA) were delivered through drinking water from day 1 to 35 at a dosage of 50 mg/L. The formulation included *B. subtilis* (5.98 × 10^9^ colony-forming unit [CFU]/g) and *B. pumilus* (1.50 × 10^9^ CFU/g), totaling 7.48 × 10^9^ CFU/g, as indicated on the product label.

### Data collection

#### Performance parameters

Feed intake

FI was recorded weekly on days 1, 8, 15, 22, 29, and 36 at 8:30 AM. FI was calculated as the difference between the feed offered and feed remaining in each replicate, using a digital scale (measured in grams).

Body weight

Body weights were measured weekly from day 1 to 36 at 9:30 AM. Two birds per replicate were also weighed individually on day 36. Initial body weight (IBW), final body weight (FBW), and body weight gain (BWG) were subsequently calculated.

Feed conversion ratio (FCR)

FCR was computed weekly by dividing total FI by BWG for the corresponding period.

#### Physiological parameters

Rectal temperature (Tr) and respiratory rate (RR)

Tr and RR were measured twice daily (10:00 AM and 12:00 PM) during the HS period (days 31–35). Two randomly selected and tagged birds per pen (12 birds/treatment) were used for repeated measurements using a digital thermometer (GLA M700, San Luis Obispo, CA). The same birds were used for RR assessments.

#### Carcass weight and organ sampling

On day 35, two birds per replicate (12 per treatment) were euthanized by jugular vein incision for blood sampling and organs collection. Carcass weight was measured post-evisceration (after wing removal). Weights of the liver, spleen, heart, and bursa of Fabricius were also recorded.

Intestinal histology

Jejunal and ileal samples were collected to evaluate intestinal morphology. Segments were harvested 10 cm proximal to Meckel’s diverticulum (jejunum) and 10 cm distal to the ileocecal junction (ileum), flushed with phosphate-buffered saline, and fixed in 10% neutral buffered formalin. Histological processing and Hematoxylin and Eosin staining were conducted at the pathology laboratory of the School of Medicine, following established protocols [21].

Blood metabolites and inflammatory biomarkers

On day 35, blood samples were collected from euthanized birds (n = 12 per treatment), centrifuged at 1,000× *g* for 15 min, and the plasma aliquots were stored at −20°C. Levels of serum amyloid A (SAA), diamine oxidase (DAO), tumor necrosis factor-alpha (TNF-α), glucose, albumin, and total protein were quantified using commercial enzyme-linked immunosorbent assay kits, following manufacturer protocols (ELK Biotechnology Co., Ltd., Wuhan, China for TNF-α, DAO, and SAA; MTD Diagnostics, Italy, for glucose, albumin, and total protein). Intra-assay coefficients of variation were 7.02%, 9.47%, 8.82%, 2.03%, 3.00%, and 2.48%, respectively.

### Statistical analysis

All statistical analyses were conducted using SAS version 9.4 (SAS Inst. Inc., Cary, NC, USA). Performance metrics, Tr, and RR were analyzed using repeated measures in the PROC MIXED procedure. The statistical model included treatment, day, and their interaction. Each replicate served as the experimental unit. The same procedure was used to analyze intestinal morphology, blood metabolites, and organ weights. Pre-planned contrasts were used to evaluate the effects of nutritional treatment (control [CON] vs. PRO) and environmental condition (TN vs. HS) [11]. Least square means were reported, with significance declared at p ≤ 0.05 and trends noted when 0.05 < p ≤ 0.10.

## RESULTS

### Physiological parameters

#### Rectal temperature

**Figure 2:**
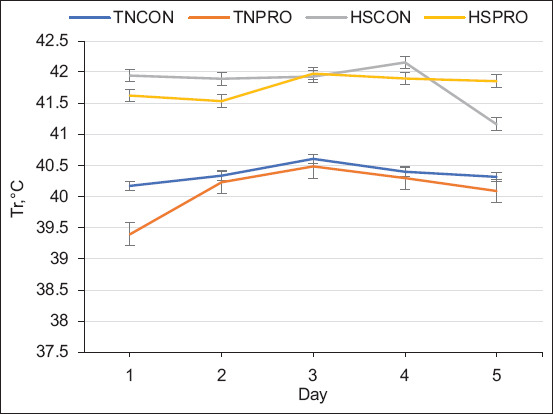
The effect of water-added probiotics on rectal temperature in broiler chickens exposed to heat stress challenge. Treatments: TNCON=Thermo-neutral control; TNPRO=Thermo-neutral+water-added probiotics; HSCON=Heat stress control; HSPRO=Heat stress+water-added probiotics. Each line represents the mean of rectal temperature over the study duration, with error bars denoting standard error of the mean.

Broilers subjected to HS conditions exhibited significantly higher Tr compared to those reared under TN conditions, with an increase of 1.6°C (p < 0.01; Figure 2). Regardless of environmental conditions, PRO supplementation in drinking water led to a significant reduction in Tr by 0.17°C (p < 0.01; Figure 2) relative to non-supplemented groups. *Post hoc* analysis further revealed that Tr in the HSPRO group was lower than in HSCON birds by 0.36°C on day 2 and 0.26°C on day 4 of the HS period.

#### Respiration rate

**Figure 3:**
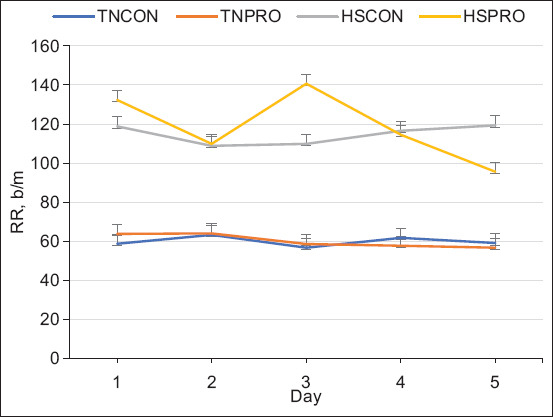
The effect of water-added probiotic on respiratory rate in broiler chickens to heat stress challenge. Treatments: TNCON=Thermo-neutral control; TNPRO=Thermo-neutral+water-added probiotics; HSCON=Heat stress control; HSPRO=Heat stress+water-added probiotics. Each line represents the mean of respiratory rate over the study duration, with error bars denoting standard error of the mean.

Broilers exposed to HS exhibited a significantly elevated RR compared to those in TN conditions, with an increase of 57 breaths/min (p < 0.01; Figure 3). Although no significant difference in RR was observed between HSPRO and HSCON groups (p = 0.21), *post hoc* analysis indicated a notable reduction in RR by 24 breaths/min in the HSPRO group on day 5 of heat exposure (p < 0.01; Figure 3).

### Carcass weight and organ samples

No significant differences were detected in carcass weight, absolute liver weight, spleen weight, or dressing percentage across treatment groups (p > 0.05; Table 2). However, liver weight as a percentage of body weight was significantly higher in HS broilers compared to TN birds (10.2%, p = 0.04; Table 2). HS conditions also tended to reduce heart weight by 8.8% relative to TN conditions (p = 0.07). The weight and percentage of the bursa of Fabricius were significantly lower in the TNPRO, HSCON, and HSPRO groups compared to TNCON (41.4%, 37.6%, 23.9% for weight; 39.3%, 32.1%, 18% for percentage; p < 0.01; Table 2).

**Table 2:** Effects of water-added probiotics on dressing percentage and organ weights in broiler chickens under thermo-neutral and heat stress conditions.

Parameter	Treatments^1^				SEM	p-value	Contrasts^2^		
	
	TNCON	TNPRO	HSCON	HSPRO			TN versus HS	CON versus PRO	HSCON versus HSPRO
Body weight (kg)	2.58	2.51	2.46	2.38	0.08	0.41	0.14	0.40	0.53
Carcass weight (g)	1731	1654	1631	1640	62	0.66	0.37	0.59	0.92
Dressing (%)	67.09	65.72	66.56	67.11	1.04	0.76	0.68	0.70	0.71
Liver weight (g)	55.13	53.77	58.78	58.17	2.96	0.58	0.18	0.74	0.89
Liver (%)	3.19	3.29	3.59	3.63	0.17	0.21	0.04	0.71	0.90
Spleen weight (g)	3.22	2.15	2.78	2.87	0.36	0.23	0.69	0.19	0.86
Spleen (%)	0.19	0.13	0.17	0.18	0.02	0.19	0.49	0.17	0.84
Heart weight (g)	14.23	13.45	13.35	11.88	0.66	0.10	0.07	0.10	0.12
Heart (%)	0.82	0.80	0.81	0.73	0.03	0.19	0.21	0.12	0.07
Fabricius gland weight (g)	4.76^a^	2.79^b^	2.97^b^	3.62^b^	0.34	<0.01	0.16	0.06	0.18
Fabricius gland (%)	0.28^a^	0.17^b^	0.19^b^	0.23a^b^	0.02	0.01	0.55	0.22	0.21

^a-c^Means with different superscripts denote an overall treatment difference (p ≤ 0.05).

1 TNCON=Thermoneutral control; TNPRO=Thermoneutral + water-added probiotics; HSCON=Heat stress control; HSPRO=Heat stress + water-added probiotics.

2 Contrasts statement: TN=TNCON + TNPRO; HS=HSCON + HSPRO. SEM=Standard error of the mean, HS=Heat stress, TN=Thermo-neutral, PRO=Probiotic, CON=Control

### Intestinal morphology

**Figure 4:**
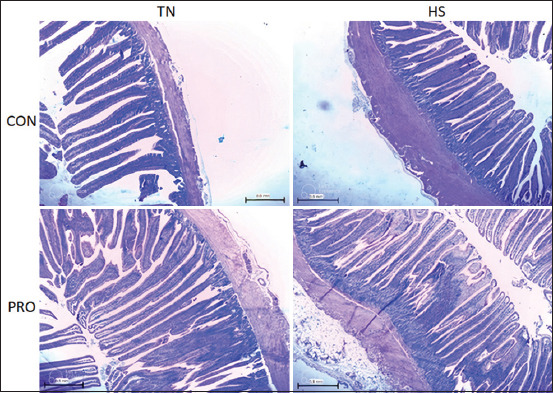
Effects of water-added probiotics on the jejunum morphology of broiler chickens on d 35. Sections were stained by hematoxylin and eosin. 40×. Treatments: TNCON=Thermo-neutral control; TNPRO=Thermo-neutral + water-added probiotics; HSCON=Heat stress control; HSPRO=Heat stress + water-added probiotics.

**Figure 5:**
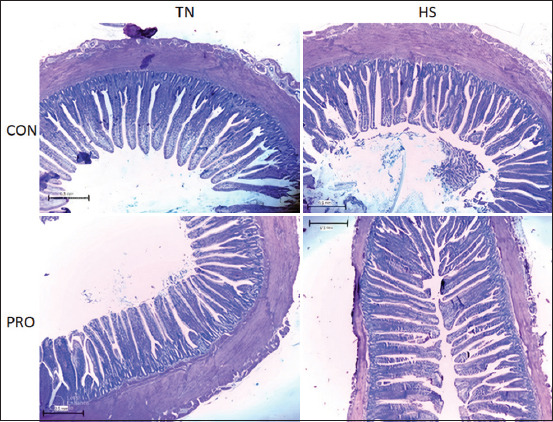
Effects of water-added probiotics on the ileum morphology of broiler chickens on d 35. Sections were stained by hematoxylin and eosin. 40×. Treatments: TNCON=Thermo-neutral control; TNPRO=Thermo-neutral + water-added probiotics; HSCON=Heat stress control; HSPRO=Heat stress + water-added probiotics.

HS significantly reduced villus height (VH) and surface area in the jejunum compared to TN treatments (p < 0.05) and tended to decrease crypt depth (CD) by 14.5%, 27%, and 6%, respectively (Table 3 and Figure 4). Similarly, HS led to reductions in VH, villus width, and CD in the ileum while increasing the VH: CD ratio by 8%, 10%, 19%, and 11%, respectively (p < 0.05; Table 3 and Figure 5). Under HS conditions, PRO supplementation significantly increased VH in both the jejunum and ileum compared to the HSCON group (by 17% and 12%, respectively; p < 0.05; Table 3; Figures 4 and 5).

**Table 3:** Effects of water-added probiotics on intestinal morphology in broiler chickens under thermoneutral and heat stress conditions.

Parameter	Treatments^1^				SEM	p-value	Contrasts^2^		
	
	TNCON	TNPRO	HSCON	HSPRO			TN versus HS	CON versus PRO	HSCON versus HSPRO
Jejunum									
Villus height (mm)	1.31^a^	1.23^a^	1.00^c^	1.17^b^	0.04	<0.01	<0.01	0.29	0.01
Villus width (mm)	0.15	0.15	0.15	0.14	0.02	0.69	0.45	0.73	0.34
Crypt depth (mm)	0.16	0.15	0.15	0.14	0.01	0.19	0.06	0.20	0.23
VH: CD	8.85	8.51	8.32	8.58	0.46	0.87	0.62	0.95	0.68
Surface area (mm^2^)	0.61^a^	0.60^a^	0.42^b^	0.48^b^	0.03	<0.01	<0.01	0.49	0.26
Ileum									
Villus height (mm)	0.92^a^	0.86^a^	0.77^b^	0.86^a^	0.03	0.02	0.03	0.64	0.08
Villus width (mm)	0.16^a^	0.14^b^	0.14^b^	0.13^b^	0.01	<0.01	0.02	0.006	0.18
Crypt depth (mm)	0.17^a^	0.15^b^	0.13^bc^	0.13^bc^	0.01	<0.01	<0.01	0.019	0.41
VH:CD	5.95	6.23	6.58	6.91	0.29	0.08	0.02	0.28	0.41
Surface area (mm^2^)	0.36	0.38	0.35	0.35	0.02	0.80	0.53	0.64	0.95

^a-c^Means with different superscripts denote an overall treatment difference (p ≤ 0.05).

1 TNCON=Thermo-neutral control, TNPRO=Thermo-neutral + water-added probiotics, HSCON=Heat stress control, HSPRO=Heat stress + water-added probiotics.

2 Contrasts statement: TN=TNCON + TNPRO; HS=HSCON + HSPRO. SEM=Standard error of the mean, VH=Villus height, CD=Crypt depth, HS=Heat stress, TN=Thermo-neutral, PRO=Probiotic, CON=Control

### Blood metabolites and inflammatory biomarkers

Albumin levels were significantly lower in HS-treated birds compared to TN birds (11.76% reduction; p = 0.02; Table 4). However, no significant differences in albumin levels were observed between PRO and CON groups, regardless of thermal condition (p = 0.76). Total DAO levels did not significantly differ among treatments (p = 0.41), although PRO supplementation tended to reduce DAO concentrations by 24%, independent of environmental conditions (p = 0.10; Table 4).

**Table 4:** Effect of water-added probiotics on blood metabolites and inflammatory biomarkers in broiler chickens under thermo-neutral and heat stress conditions.

Parameter	Treatments^1^				SEM	p-value	Contrasts^2^		
	
	TNCON	TNPRO	HSCON	HSPRO			TN versus HS	CON versus PRO	HSCON versus HSPRO
Albumin (g/dL)	0.92	0.94	0.85	0.79	0.04	0.08	0.02	0.76	0.37
DAO^3^ (ng/mL)	9.70	7.63	9.82	7.19	1.37	0.41	0.91	0.9	0.19
Glucose (mg/dL)	291^a^	261^b^	243^c^	185^d^	4.74	<0.01	<0.01	<0.01	<0.01
SAA^4^ (µg/mL)	0.69	0.69	0.67	0.60	0.07	0.72	0.41	0.61	0.41
TNF-α^5^ (pg/mL)	46.10	39.35	40.24	29.84	6.89	0.45	0.28	0.23	0.24
Total protein (g/dL)	3.29	3.02	3.04	3.11	0.12	0.36	0.50	0.39	0.70

^a-c^Means with different superscripts denote an overall treatment difference (p ≤ 0.05).

1 TNCON=Thermoneutral control, TNPRO=Thermoneutral + water-added probiotics, HSCON=Heat stress control, HSPRO=Heat stress + water-added probiotics.

2 Contrasts statement: TN=TNCON + TNPRO; HS=HSCON + HSPRO.

3 Diamine oxidase.

4 Serum amyloid A.

5 Tumor necrosis factor-alpha. SEM=Standard error of the mean, HS=Heat stress, TN=Thermo-neutral, PRO=Probiotic, CON=Control

During the heat challenge, glucose levels were significantly reduced in HS broilers by 22.5% compared to TN birds (p < 0.01; Table 4). Furthermore, HSPRO birds exhibited a 24.0% reduction in glucose levels compared to HSCON birds (p < 0.01). No significant treatment-related differences were found in SAA, TNF-α, or total protein levels (p > 0.05; Table 4).

### Performance results

**Figure 6:**
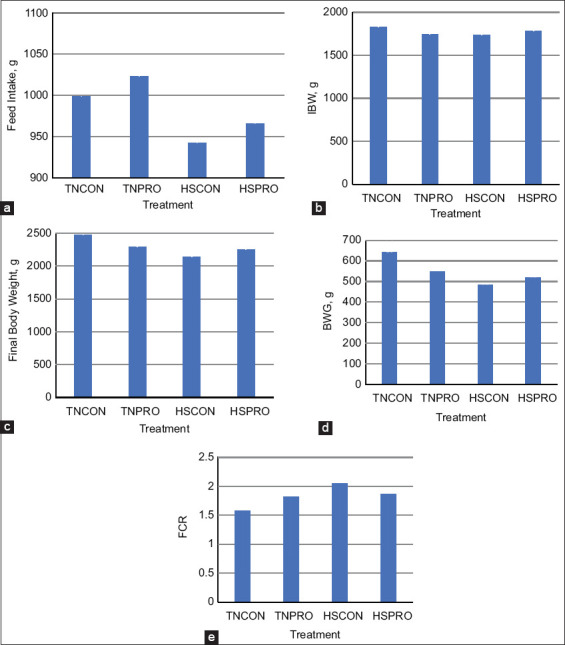
The effect of water-added probiotics on (a) feed intake, (b) initial body weight (IBW), (c) final body weight, (d) body weight gain, and (e) feed conversion ratio in broiler chickens over 4 weeks. TNCON=Thermo-neutral control; TNPRO=Thermo-neutral+water-added probiotics; HSCON=Heat stress control; HSPRO=Heat stress+water-added probiotics. Each bar represents the mean IBW over the study duration, with error bars denoting standard error of the mean.

During the 5**^th^** week, coinciding with the HS challenge, neither environmental conditions (HS vs. TN) nor PRO supplementation significantly affected FI, IBW, FBW, BWG, or FCR. Performance metrics remained comparable between CON and PRO groups (p > 0.05; Figure 6).

## DISCUSSION

High ambient temperatures serve as a major stressor in poultry production, markedly impairing growth performance, egg production, product quality, and survivability [1]. Exposure to HS has been shown to reduce productivity, compromise intestinal integrity, and facilitate increased bacterial translocation into the bloodstream in poultry [7, 22]. The resultant increase in gut permeability facilitates the passage of harmful bacteria and toxins into the host’s circulatory system, thereby exacerbating inflammatory immune responses [23]. These adverse effects underscore the significant impact of HS on poultry health and productivity, highlighting the urgent need for effective mitigation strategies [24].

Maintaining optimal growth performance in broiler chickens is essential for sustaining the efficiency and profitability of poultry enterprises. The method of PRO administration influences both growth performance and immune modulation, with supplementation through drinking water demonstrating greater efficacy than in-feed delivery [25]. Water-based PRO administration is often favored due to its higher absorption efficiency and easier integration into poultry management systems, potentially reducing labor requirements [19]. In the present study, we evaluated the effects of water-supplemented PROs on broiler performance and physiological responses under both TN and HS conditions.

Broilers perform optimally when maintained within their TN zone (TNZ), typically ranging between 18°C and 25°C [24]. The average core body temperature of chickens is generally between 41°C and 42°C [18]. In response to temperatures exceeding the TNZ, broilers – being homeothermic – exhibit elevated body temperatures, making Tr a reliable indicator of thermal stress [24]. Under HS, chickens struggle to dissipate excess heat due to their limited thermoregulatory mechanisms, resulting in increased Tr [26]. The observed 1.6°C elevation in Tr under HS conditions corroborates earlier reports of HS-induced thermoregulatory activation. Notably, PRO supplementation led to a significant reduction in Tr by 0.17°C, regardless of ambient conditions, with the most pronounced decreases observed on days 2 and 4 of the heat challenge in the HSPRO group. These findings align with findings of Aluwong *et al*. [27], who reported reduced Tr in broilers administered PROs under HS. Conversely, Abdelqader *et al*. [28] found no significant effect of PROs on Tr, even under HS conditions (30°C ± 1°C), suggesting that strain type, dosage, and environmental severity may modulate the PRO response. Our data suggest that water-supplemented PROs may contribute to thermotolerance by modulating metabolic or physiological pathways involved in heat dissipation.

Unlike mammals, birds lack sweat glands and instead rely on behavioral and physiological adaptations – such as panting, wing spreading, and increased RR – to cope with elevated temperatures [29]. RR is a useful physiological indicator of HS severity and its impact on acid-base balance. In this study, HS significantly increased RR by 57 breaths/min compared to TN conditions, consistent with previous findings by Gogoi *et al*. [30]. Although no statistically significant difference in RR was observed between HSPRO and HSCON treatments overall, a reduction of 24 breaths/min on day 5 in HSPRO birds suggests a potential late-phase PRO effect on respiratory stress mitigation.

Despite measurable improvements in Tr and RR, PRO supplementation had no significant effects on key performance indicators – FI, IBW, FBW, BWG, or FCR – under either TN or HS conditions. These results are consistent with Li *et al*. [31], who reported no improvements in broiler performance following PRO administration under both normal and heat-stressed conditions. Similar findings were reported by Van Der Klein *et al*. [32], who observed no significant differences in FI, BW, or FCR during HS, and by Shargh *et al*. [33], who found that PROs did not enhance FI or BWG in TN environments. In contrast, Biswas *et al*. [34] reported increased BWG and reduced FCR in PRO-treated broilers, though without changes in FI. Such inconsistencies may stem from variations in PRO strain, dosage, broiler genotype, and environmental stress intensity. Thus, under the current study conditions, neither HS nor PRO supplementation significantly influenced broiler growth performance.

The assessment of carcass and organ traits offers additional insight into physiological responses to environmental and dietary interventions. HS has been associated with oxidative damage and lymphoid organ atrophy [18]. PROs may attenuate these effects by reducing corticosterone levels, thereby preserving lymphoid organ mass [35]. However, in the present study, body weight, carcass weight, dressing percentage, and organ weights (liver, spleen, and heart) did not differ significantly across treatments, indicating no effect of PROs or HS on these traits. The exception was liver weight as a percentage of body weight, which was significantly elevated in HS birds. This finding is consistent with Ma *et al*. [3], who attributed liver hypertrophy to fat accumulation and compensatory mechanisms under thermal stress.

Gut morphology plays a pivotal role in nutrient absorption and overall poultry health. An increased VH-to-CD (VH:CD) ratio is indicative of enhanced absorptive capacity [36]. HS is known to impair intestinal architecture, leading to shorter villi, deeper crypts, and reduced absorptive surface area [35, 37]. Our results confirmed these patterns, with HS significantly reducing VH and surface area in the jejunum and ileum, while increasing the VH: CD ratio in the ileum. These results align with Lan *et al*. [38], who reported similar morphological deterioration in heat-stressed broilers. Notably, PRO supplementation under HS partially reversed the villus atrophy, as observed in previous studies by Li *et al*. [31]. In contrast, Shargh *et al*. [33] found that water-administered PROs had no impact on intestinal segment dimensions in TN conditions, a finding corroborated by our data. The observed variability in intestinal histomorphology across studies may be attributed to differences in PRO strains, dosage, experimental duration, and anatomical site of assessment [39, 40].

Biochemical biomarkers offer further insight into systemic physiological responses. Our data revealed significantly lower plasma albumin and glucose levels in HS-treated broilers compared to TN counterparts, consistent with findings from Attia *et al*. [41]. While HS had no significant effect on albumin levels in Japanese quail [42], reduced glucose levels under HS conditions were also documented in laying hens [43]. Notably, the literature reveals inconsistent glucose responses – ranging from increases [44], decreases [45], or no changes [30] – likely reflecting differences in breed, age, environmental parameters, and experimental design. Early-life HS exposure may enhance thermoregulatory adaptation, as birds exposed for 4 days exhibited improved Tr and glucose levels compared to those exposed for only 2 days [1].

Gut barrier dysfunction under HS facilitates translocation of pathogens and toxins, exacerbating intestinal inflammation and elevating proinflammatory cytokine levels [46]. In our study, neither environmental condition nor PRO supplementation significantly altered SAA or TNF-α levels. This contrasts with Wu *et al*. [37], who reported increased TNF-α expression in the jejunum and ileum under HS. DAO levels tended to be lower in PRO-supplemented groups, aligning with Yu *et al*. [47], who also reported reduced DAO levels in treated birds. However, DAO concentrations did not differ significantly between HS and TN groups in our study, diverging from Lan *et al*. [38], who observed elevated DAO under HS. DAO release into circulation reflects intestinal mucosal injury, particularly from villus tip cells, and may serve as a useful biomarker of barrier integrity [37].

## CONCLUSION

This study evaluated the effects of PRO-supplemented drinking water on the physiological responses, intestinal morphology, blood biomarkers, and performance metrics of broiler chickens exposed to TN and HS conditions. The results demonstrated that HS significantly elevated Tr and RR, impaired intestinal architecture, and reduced albumin and glucose concentrations, thereby confirming the deleterious effects of thermal stress on broiler health and metabolism. PRO supplementation conferred modest thermoregulatory benefits by reducing Tr and RR, and it tended to lower DAO levels, suggesting a potential protective role in maintaining intestinal barrier function. In addition, PROs partially ameliorated HS-induced villus atrophy in the jejunum and ileum. However, the supplementation did not significantly improve growth performance parameters (FI, BWG, FCR) or inflammatory biomarkers (SAA, TNF-α) under either TN or HS conditions.

The strength of this study lies in its factorial design, which allowed simultaneous evaluation of environmental and nutritional effects under controlled laboratory conditions. The use of standardized histological, physiological, and biochemical assessments provided a comprehensive understanding of PRO efficacy in the context of HS. Furthermore, the administration of a commercially relevant *Bacillus*-based PRO through drinking water reflects practical application for poultry producers.

Nonetheless, the study has certain limitations. The HS model employed was of short duration (5 days), which may not fully capture the cumulative physiological and performance impacts of chronic HS. Moreover, the study was limited to a single PRO formulation and dosage, precluding the evaluation of dose-dependent or strain-specific effects. The absence of microbiota profiling also restricted mechanistic insights into how PROs modulate gut health under stress.

Future research should investigate the long-term effects of PRO supplementation under prolonged or cyclic HS conditions. Studies exploring strain combinations, varied dosages, and delivery methods (e.g., microencapsulation or *in ovo* administration) may enhance the efficacy of PRO interventions. Integration of omics-based approaches such as gut microbiome sequencing, transcriptomics, and metabolomics would also provide deeper insights into host-microbe interactions and mechanisms of action. In addition, evaluating synergistic combinations of PROs with other HS amelioration strategies – such as phytochemicals, organic acids, or antioxidants – could contribute to the development of integrated nutritional approaches to improve poultry resilience in the face of climate change.

## AUTHORS’ CONTRIBUTIONS

RI, MA, and MAQ: Conceptualized, methodology, analyzed and interpreted the data, and drafted the manuscript. RI, MA, MAQ, MAAM, and AA: Supervised the study and critically revised the manuscript for important intellectual content. All authors have read and approved the final manuscript.
